# Synthesis and Application of Arylaminophosphazene as a Flame Retardant and Catalyst for the Polymerization of Benzoxazines

**DOI:** 10.3390/polym13020263

**Published:** 2021-01-14

**Authors:** Natalia V. Bornosuz, Irina Yu. Gorbunova, Vyacheslav V. Kireev, Yulya V. Bilichenko, Larisa V. Chursova, Yuri S. Svistunov, Denis V. Onuchin, Vyacheslav V. Shutov, Viktoria V. Petrakova, Alexander A. Kolenchenko, Duong T. Nguyen, Nikolay V. Pavlov, Alexey V. Orlov, Tatyana A. Grebeneva, Igor S. Sirotin

**Affiliations:** 1 Mendeleev University of Chemical Technology of Russia, 125047 Moscow, Russia; bornosuz@muctr.ru (N.V.B.); igorbunova@muctr.ru (I.Y.G.); kireev@muctr.ru (V.V.K.); bilichenko@muctr.ru (Y.V.B.); donuchin@muctr.ru (D.V.O.); shutov1105@gmail.com (V.V.S.); vvvorobeva@muctr.ru (V.V.P.); kolenchenkoalex@muctr.ru (A.A.K.); nguyentanyen2@gmail.com (D.T.N.); npavlov@muctr.ru (N.V.P.); 2 Prepreg-ACM JSC (Part of UMATEX, Rosatom State Corporation), 109316 Moscow, Russia; l.chursova@umatex.com (L.V.C.); a.orlov@umatex.com (A.V.O.); t.grebeneva@umatex.com (T.A.G.); 3 NPK Khimprominzhiniring, JSC (Part of UMATEX, Rosatom State Corporation), 109316 Moscow, Russia; y.svistunov@umatex.com

**Keywords:** benzoxazines, phosphazenes, curing kinetics, flammability, flame retardant, catalysis, m-toluidine

## Abstract

A novel type of phosphazene containing an additive that acts both as a catalyst and as a flame retardant for benzoxazine binders is presented in this study. The synthesis of a derivative of hexachlorocyclotriphosphazene (HCP) and meta-toluidine was carried out in the medium of the latter, which made it possible to achieve the complete substitution of chlorine atoms in the initial HCP. Thermal and flammability characteristics of modified compositions were investigated. The modifier catalyzes the process of curing and shifts the beginning of reaction from 222.0 °C for pure benzoxazine to 205.9 °C for composition with 10 phr of modifier. The additive decreases the glass transition temperature of compositions. Achievement of the highest category of flame resistance (V-0 in accordance with UL-94) is ensured both by increasing the content of phenyl residues in the composition and by the synergistic effect of phosphorus and nitrogen. A brief study of the curing kinetics disclosed the complex nature of the reaction. An accurate two-step model is obtained using the extended Prout–Tompkins equation for both steps.

## 1. Introduction

Nowadays, it is impossible to imagine human life without polymer matrix composite materials (PMCs). The unique combination of polymer matrix and filler works together to provide it unique properties; the key among them are high physical and operational characteristics, often exceeding the values of traditional materials (metals and their alloys, wood) in a wide temperature range, with low specific gravity combined with high strength, moisture and chemical resistance, radio transparency, excellent dielectric properties, durability, resistance to significant cyclic loads, etc. [1,2,3]. The abovementioned properties allowed polymer composite materials to take their place in our daily life; PMCs are used in automotive and shipbuilding industries, aircraft, sports, medical applications and many other fields [4,5,6]. One of the main disadvantages of most polymers and many composites based on them is their high flammability due to the organic nature of the matrix, which limits wider application of PMCs.

The common way to reduce the flammability of polymer composites is to introduce flame retardants, which can be divided into two groups: additive and reactive [7,8]. Additive flame retardants are not capable of chemically binding to the polymer matrix of the composition; therefore, either the phenomenon of exfoliation of the flame retardant or the phase inversion binder-modifier may occur, and, as a result, deterioration of the physical, mechanical and operational properties of products may take place. Reactive, as opposed to additive, flame retardants have functional groups in their structure that can interact with the reaction centers of the binder during the curing process and thereby incorporate into the polymer matrix network. The application of these flame retardants reduces the flammability of the material and could increase mechanical properties. 

Phosphorus-containing compounds are widespread flame retardants. Phosphazene-based retardants can be either additive or reactive. The main chain of organophosphazenes consists of alternating phosphorus and nitrogen atoms, and at the phosphorus atom, there are organic radicals introduced by the substitution of halogen in halogen phosphazenes. The nature of organic substituents can vary within wide limits and determines the properties of the resultant polymer. For example, aryloxyphosphazenes, in comparison with other organophosphorus compounds, have higher thermal stability and chemical resistance and are known as fire retardants [8,9], characterized by the synergistic effect of phosphorus and nitrogen [10]. The other example is hexaphenoxycyclotriphosphazene, which is used as a highly effective flame retardant for a large number of synthetic polymers. A number of effective flame-retarded functional epoxy resins, amine curing agents and benzoxazines with a phosphazene core have been proposed [11,12,13,14,15,16,17,18,19,20,21]. Carboxyl-containing epoxyphosphazenes and their use as a catalyst for the polymerization of benzoxazines have also been reported [22].

Benzoxazine monomers, as a novel class of binders, are currently commercialized by many leading manufacturers, such as Huntsman Advanced Materials, Kaneka Aerospace, Gurit, etc. Due to the special molecular structure of benzoxazine and its corresponding outstanding properties, five different types of benzoxazine binders are present in the market for aerospace, electronic or civilian use.

The high curing temperature of benzoxazines is one of the problems that limits their widespread application [23]. The main way to solve this problem is to use catalysts [24,25,26,27,28,29,30,31,32,33] such as phenols [24,25], strong acids [26], carboxylic acids [27,28] and Lewis acids [29,30,31,32,33]. All listed compounds are effective catalysts for the homopolymerization of benzoxazines; however, due to their high catalytic efficiency, one cannot use them in a mixture with the most common reactive compounds, such as epoxy resins, since they cause earlier curing of epoxy oligomers, thereby complicating the polymerization of benzoxazine. Among other things, the use of carboxylic acids as catalysts is complicated by the process of decarboxylation at high temperatures, which is accompanied by the release of carbon dioxide, and, as a rule, an increase in the porosity of the composition during curing.

Base catalysts such as amines, imidazoles and organophosphorus compounds, which exhibit a weaker catalyzing effect in comparison with acidic ones, are less applicable. Although commercial polybezoxazines are effective flame retardants in comparison with other polymers, they usually do not reach the UL-94 V-0 rating, so the development of halogen-free flame retardants that are well compatible with base benzoxazine is an urgent task. Variability in the molecular design of phosphazenes allows one to use them as a base of new flame retardants for benzoxazines. However, the known phosphazene components are difficult to synthesize, and, therefore, their industrial applicability may be limited. Thus, the unique properties and widely variable molecular design of organophosphazene allowed us to use it in our work as a base of a flame retardant and catalyst.

This paper describes the synthesis of a new halogen-free phosphorus-containing flame retardant based on hexachlorocyclotriphosphazene and m-toluidine, its effect on the kinetics of thermal curing and the flammability of a benzoxazine polymer. It is assumed that in addition to low flammability, the resulting phosphazene can catalyze the thermal curing of benzoxazines due to the presence of secondary hydrogen atoms in the m-toluidine residue of phosphazene in its structure.

## 2. Materials and Methods 

### 2.1. Starting Materials

Hexachlorocyclotriphosphazene—a white crystalline substance with m.p. of 113 °C; nuclear magnetic resonance (NMR) ^31^P-singlet spectrum with δ_P_ = 19.9 ppm was obtained by the method in [34]. M-toluidine (Acros Organics, Geel, Belgium), a colorless liquid, was distilled twice under vacuum before use, b.p. 203–204 °C. Solvents were purified according to known methods, and their physical characteristics corresponded to literature data [35].

### 2.2. Synthesis of Hexakis-(3-methylphenylamino)cyclotriphosphazene (PN-mt)

A three-necked 500 mL round bottom flask equipped with a top-drive agitator, reflux condenser and an inert gas supply system was charged with 25.00 g (0.072 mol) of hexachlorocyclotriphosphazene (HCP) and 247.25 g of m-toluidine (2.311 mol). The reaction mixture was refluxed under intensive stirring at a temperature of 100 °C for 1 h; after that, the temperature was increased to 180 °C and refluxed for the next 2 h. Then, the excess of m-toluidine was distilled off using a vacuum rotary evaporator. First, the remaining dark gray mass was repeatedly washed with 0.1 M aqueous solution of hydrochloric acid and washed until neutral. The resulting product was dried in a vacuum until a constant mass was achieved. The product was a gray crystalline powder. The yield was 44.02 g (80%).

### 2.3. Preparation of Benzoxazine Monomer Based on Bisphenol A, M-Toluidine and Paraphormaldehyde (BA-mt)

The benzoxazine monomer BA-mt (bis(3-(m-tolyl)-3,4-dihydro-2H-1,3-benzoxazine) was selected as the main component of the composition. It was synthesized using the method reported in the literature [36]. In contrast to the commercially available monomer BA-a (bis (3-phenyl-3,4-dihydro-2H-1,3-benzoxazine)), which has a glass transition temperature of 173 °C, BA-mt is characterized by 217 °C for completely cured samples by mode 2 h 180 °C, 4 h 200 °C, 1 h 210 °C.

### 2.4. Composition Preparation

In order to determine the effect of the synthesized phosphazene compound on the flammability and thermal properties of benzoxazine, the compositions presented in Table 1 were prepared and studied.

The calculated amount of BA-mt and PN-mt was mixed on a magnetic stirrer at 120 °C for 10 min to achieve a uniform distribution of powdered PN-mt in the benzoxazine. Subsequent degassing of the systems was performed at 120 °C for 15 min at a residual pressure of 1.0 kPa. At the end of the degassing process, the resulting compositions either were used as received for the curing study or were cured at 180 °C for 6 h for glass transition and flammability measurements.

### 2.5. Measurements

The ^1^H, ^31^P and ^13^C NMR spectra were obtained in DMSO-d6 solutions with a Bruker AV-600 spectrometer (Bruker Corporation, Bremen, Germany) operating at 600, 133 and 81 MHz, respectively. The signals, due to the deuterated solvents, were used as internal references. The chemical shifts of the signals were calculated relative to the signals of tetramethylsilane (^1^H, ^13^C) and phosphoric acid (^31^P), which were used as references. The spectra were processed with the help of the MestReNova Lab software package (version 12.0.4, MESTRELAB RESEARCH, S.L, Santiago de Compostela, Spain).

Differential scanning calorimeter DSC 204 F1 Phoenix (Netzsch, Selb, Germany) was used for monitoring the curing kinetics in dynamic mode [37]. The temperature characteristics of the curing and glass transition temperatures of cured samples were determined according to ISO 11357-5:1999 [38] and ISO 11357-2:1999 [39], respectively. The heating rate for all measurements was 10 °C/min. All tests were performed in the temperature range 50–300 °C in a nitrogen atmosphere at a rate flow of 50 mL/min. The weight of the samples ranged from 5 to 10 mg. For data processing, Proteus Thermal Analysis version 5.2.1. software (Netzsch, Selb, Germany) was used.

Fourier transform IR (FTIR) spectra were recorded on a Magna-IR-750 spectrometer Nicolet FTIR (Labx, Midland, ON, Canada) Center using KBr pellets. The spectra were processed using the Omnic software version 9.2.8.6 Thermo Fisher Scientific Inc (LabX, Midland, ON, Canada).

An elemental analyzer for sulfur, chlorine, nitrogen and carbon Multi EA 5000 was used.

Flammability tests were carried out in accordance with UL-94 [40]; the size of the samples was 127 mm × 12.7 mm × 2 mm.

## 3. Results and Discussion

One of the main disadvantages of benzoxazines that limits their use is a long and “hard” curing mode, whose temperatures can reach 220 °C. To achieve this temperature, special expensive high-temperature equipment is required in industry, which significantly complicates and increases the cost of benzoxazine binder processing. In practice, the maximum temperature for polymer matrix composite (PMC) molding in manufacturing is about 180 °C or less. 

Thus, it becomes relevant to search for the possibility to reduce the final curing temperature of a new type of phenol-formaldehyde oligomer to a temperature of no more than 180 °C with the achievement of maximum performance characteristics. That is why PN-mt seems to be a unique and the most versatile modifier of its kind.

### 3.1. Synthesis and Characteristics of Hexakis-(3-methylphenylamino)cyclotriphosphazene (PN-mt)

**Figure 1:**
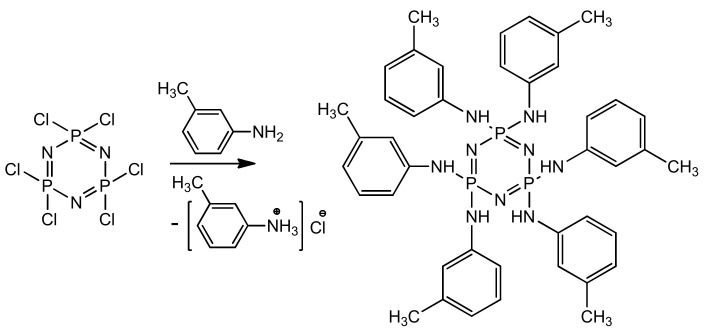
Synthesis of hexakis-(3-methylphenylamino)cyclotriphosphazene.

**Figure 2:**
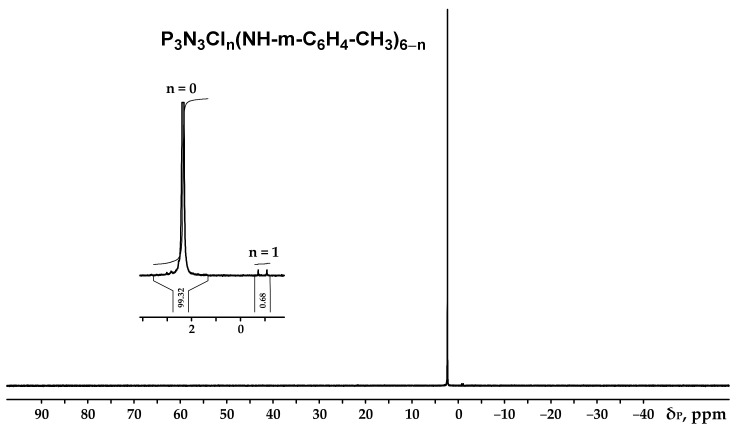
NMR ^31^P spectrum of hexa-(m-toluidine)cyclotriphosphazene (PN-mt).

**Figure 3:**
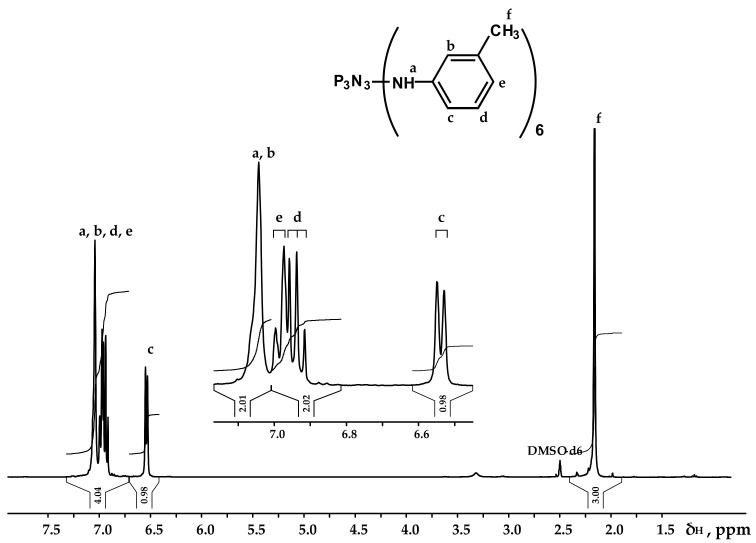
NMR ^1^H spectrum of PN-mt.

**Figure 4:**
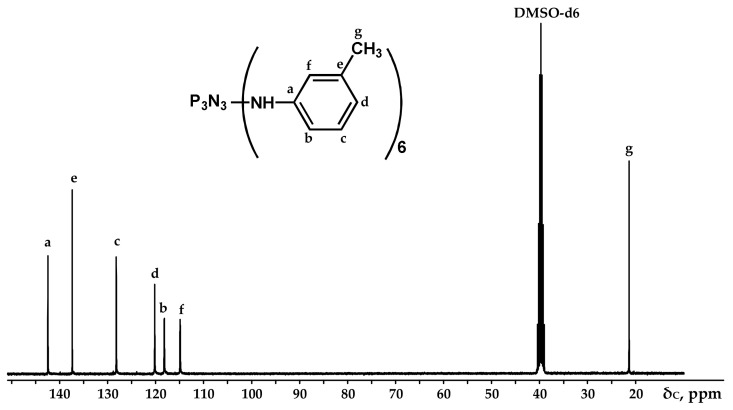
NMR ^13^C spectrum of PN-mt.

**Figure 5:**
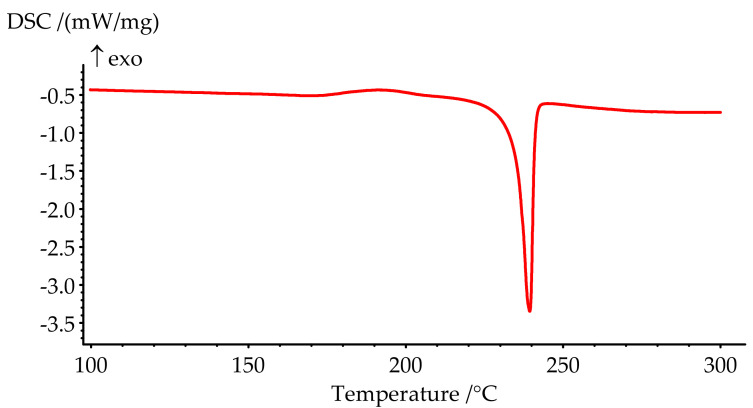
DSC curve of PN-mt melting.

Preparation of PN-mt was carried out according to the following scheme (Figure 1):

The obtained product was characterized by ^31^P, ^1^H and ^13^C NMR spectroscopy (Figure 2, Figure 3 and Figure 4), differential scanning calorimetry (Figure 5) and elemental analysis (Table 2).

It can be seen from the ^31^P NMR spectrum (Figure 2) that all the chlorine atoms in the phosphazene cycle were completely replaced by m-toluidine. Analyzing the ^1^H NMR spectrum, it can be assumed that the signal of the proton of the amine group is superimposed on the signal of one of the protons of the aromatic ring, giving a singlet of double intensity. Thus, we can conventionally assume that the intensity of the signals of the protons of the amine group is equal to 1 and, accordingly, the ratio of protons of the P-NH group to methyl protons of m-toluidine is 1:3 as calculated. The structure of radicals in the side chain is also confirmed by the ^13^C NMR spectrum, which contains signals of all characteristic carbon atoms (Figure 4).

The melting point of PN-mt, determined by the DSC method as a peak temperature, is 239.4 °C and presented on the DSC curve (Figure 5). The phase transition is characterized by a significant endothermic effect and equal to 84.8 J/g.

The results of the element analysis are consistent with theoretical calculations for the content of elements in the resulting compound (Table 2).

### 3.2. Synthesis and Characteristics of BA-mt

**Figure 6:**
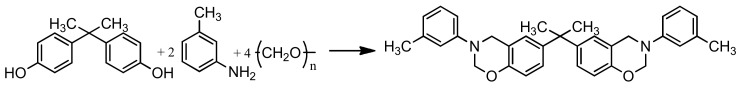
Synthesis of BA-mt.

Preparation of BA-mt was carried out according to the following scheme (Figure 6):

The synthesis was carried out in a toluene medium at the temperature of 80–90 °C with a 10% excess of paraformaldehyde. The product yield was 95%.

**Figure 7:**
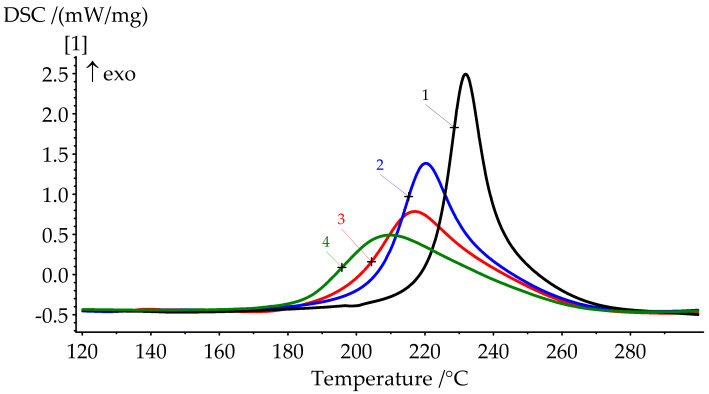
DSC curves of formulations 1–4 cure.

The obtained product was characterized by ^1^H NMR spectroscopy (Table 3) and differential scanning calorimetry (Figure 7, curve 1; Table 4, №1).

The ^1^H NMR spectrum contains signals of the oxazine ring in the region δ_H_ = 4.56 and 5.30 ppm, as well as signals of the methyl groups of aromatic amine and bisphenol a in the region δ_H_ = 2.30 and 1.57 ppm, respectively.

### 3.3. Polymerization of BA-mt in Presence of PN-mt

**Figure 8:**
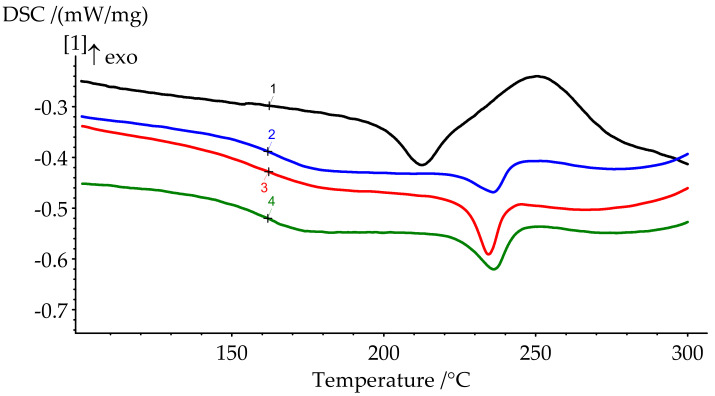
DSC curves of cured formulations 1–4.

Polymerization of BA-mt in the presence of PN-mt was monitored by DSC. Characteristic temperatures of curing and glass transition temperatures of the resulting compositions are presented in Figure 7 and Figure 8 and in Table 4.

**Figure 9:**
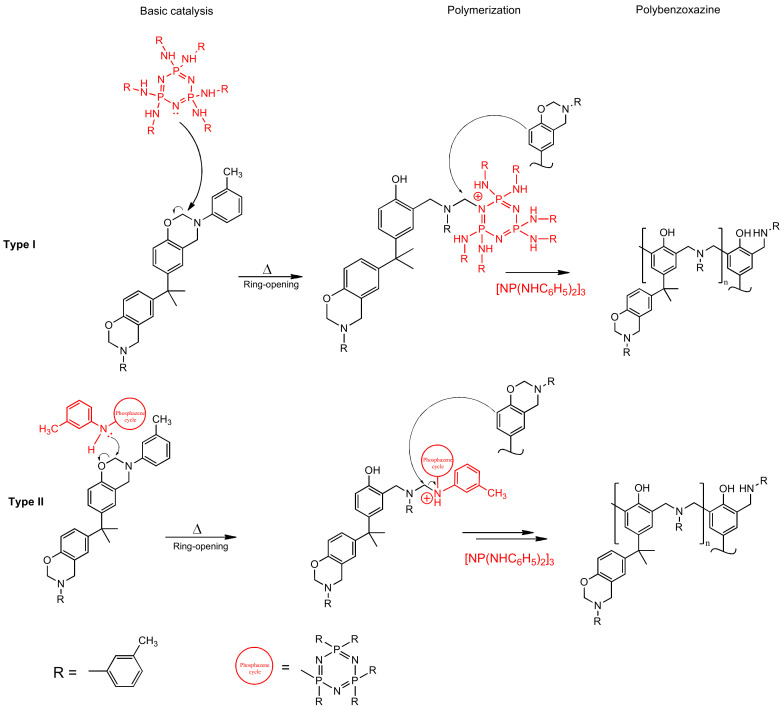
Proposed mechanism of benzoxazine polymerization catalyzed by PN-mt.

DSC results show a significant shift of the onset of curing to the lower temperatures when modifier is added. The catalytic effect is quite explicit. For 10 pbw (formulation 2), this shift appears to be 16.1 °C; for 30 pbw (formulation 4), the apparent shift is 37.2 °C. Here, we can see a decrease in the heat release during curing from 346.2 J/g for unmodified BA-mt (formulation 1) to 252.0 J/g for 30 pbw (formulation 4). This may be due to the possibility of a nucleophilic attack of the lone electron pair of the nitrogen atom in PN-mt on the methylene bridge connecting the oxygen and nitrogen atoms in the benzoxazine ring (Figure 9).

The degree of curing under the given conditions increases with an increase in the concentration of PN-mt in the compositions. The presence of residual enthalpy indicates the incomplete cure of compositions. It should be noted that an increase in the concentration of PN-mt in the mixture, starting from 10 pbw, leads to the appearance of an endothermic peak in the region of 240 °C, corresponding to the melting point of neat PN-mt. It indicates incomplete compatibility of the modifier with the matrix. This is one of the explanations for the significant decrease in the glass transition temperature when more than 10 pbw of PN-mt is added to the composition. The glass transition temperature dramatically falls from 206.5 °C for unmodified composition (formulation 1) to 164.9 °C for 10 pbw (formulation 2) and does not significantly differ within the modification. The difference in *T*_g_ between formulations 2 and 4 is only 4.8 °C.

Another reason for the decrease in the above-described characteristic is the significant size of the modifier molecules, which is comparable to nanoparticles with a diameter of 1–2 nm. It results in a change in the spatial regularity of the formed polybenzoxazine network and a decrease in the number of hydrogen bonds between hydrogen atoms in phenolic groups and nitrogen in polymerized benzoxazines.

Secondly, in contrast to the classical understanding of a catalyst, which only reduces the activation energy of the reaction without being affected by itself, the synthesized PN-mt could be simultaneously both a catalyst and a comonomer that participates in the polymerization of the benzoxazine monomer BA-mt and is then embedded in a three-dimensional structure. This effect is associated with the increased reactivity of the ortho and para positions in the m-toluidine substituent of PN-mt, due to the presence of a methyl substituent in the meta-position, which has an electron-donating effect. This assumption is intended to be investigated more thoroughly in our further study.

DSC curves of cured samples could also indicate the stability of the materials. The heat release at elevated temperatures usually corresponds to the thermal destruction. The slight rise of the heat flow appears at the end of the runs (Figure 8). Thus, we expect materials to be stable approximately up to 300 °C.

**Figure 10:**
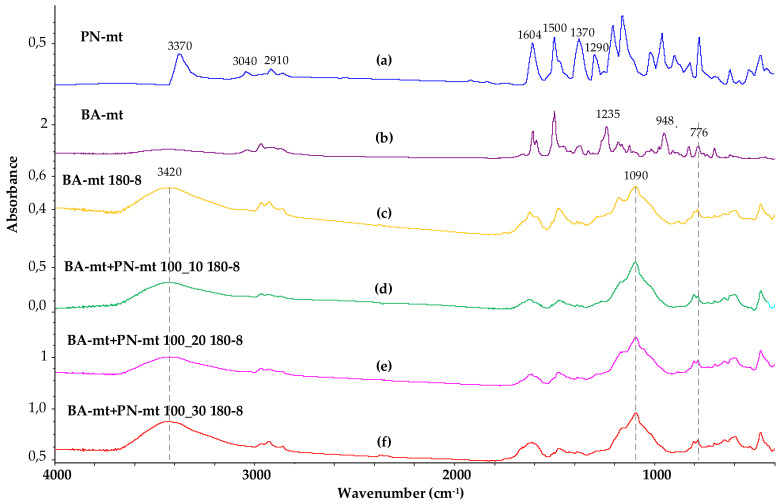
FTIR spectra of PN-mt (**a**), BA-mt monomer (**b**), cured BA-mt (**c**) and cured compositions containing 100 pbw of BA-mt and 10 (**d**), 20 (**e**) and 30 (**f**) pbw of PN-mt.

The curing process of the composition was also characterized using FTIR spectroscopy. Figure 10 shows the FTIR spectra of the initial flame retardant PN-mt and benzoxazine monomer BA-mt, as well as four samples of cured compositions, both without using a flame retardant and with its addition in various ratios.

Analyzing the FTIR spectrum of the PN-mt compound, we can conclude that it contains all characteristic signals confirming its structure: signals at 1604 and 1290 cm^−1^ correspond to vibrations of the secondary amino group associated with the benzene ring; signals at 2910 and 1370 cm^−1^ refer to stretching and bending vibrations, respectively, of methyl groups; signals at 3040, 1600 and 1500 cm^−1^ are typical for aromatic compounds. The groups of signals responsible for oscillations of the phosphazene cycle are in the range of 700–1200. The signal at 3370 is not entirely clear; it is probably related to the NH bond of PN-mt.

In the FTIR spectrum of the BA-mt monomer, the signals at 948 and 1235 cm^−1^ correspond to the stretching vibrations of the C-O-C bond, and at 752 cm^−1^, to the out-of-plane bending vibrations of Ar-H in the oxazine ring. In the infrared spectra of the obtained polybenzoxazines, the intensities of these signals are significantly reduced, and a broadened signal appears at 3420, which corresponds to the stretching vibrations of phenolic hydroxyl groups linked by hydrogen bonds. It should be noted that the addition of a fire retardant to the composition in various amounts practically does not affect the FTIR spectrum. Thus, the data of FTIR spectroscopy do not allow us to make an unambiguous conclusion about the participation of the ortho and para positions of PN-mt in the curing reaction and the formation of covalent bonds between PN-mt and BA-mt. Taking into account the presence of the residual endo-peak on the DSC curves (melting of PN-mt) of the cured modified compositions, it can be concluded that at least part of the modifier is not covalently incorporated into the polymer network.

### 3.4. Curing Kinetics

The common approach to studying curing kinetics is to monitor it by differential scanning calorimetry (DSC), as the crosslinking reaction of thermosetting resins is an exothermic process.

Two main strategies can be used to model the DSC results and derive kinetic laws: model-free and model fitting methods. Model-free isoconversional methods allow us to determine the activation energy as a function of conversion with the simple assumption that “the reaction rate at constant extent of conversion is only a function of temperature”, i.e., only for single-step reactions [37]. However, the curing mechanism of thermosetting resins is usually a multi-step one. Particularly, non-negligible contribution of the molecular diffusion might seriously hinder the kinetics of curing [41,42,43].

The degree of curing is supposed to be proportional to the amount of heat generated during the process and is usually defined as:

(1) $$\propto \left(t\right)=\;\frac{H\left(t\right)}{\Delta H_{total}}$$

where *H(t)* is the accumulative heat of reaction up to a given time *t* during the curing process; ∆*H_total_* is the ultimate total heat released at complete cure. For an uncured system, α = 0, whereas for a completely cured one, α = 1.

The curing rate is assumed to be proportional to the rate of heat generation *dα/dt* and is calculated by the following equation:

(2) $$\frac{d\propto }{dt}=\;\frac{1}{\Delta H_{total}}\frac{dH}{dt}$$

It is currently accepted that the rate of reaction can be defined by two functions, *k(T)* and *f(α)*:

(3) $$\frac{d\propto }{dt}=k\left(T\right)f\left(\propto \right)$$

where *dα/dt* is the reaction rate (s^−1^), *α* is the degree of conversation (dimensionless), *k(T)* is the temperature-dependent rate constant, and *f(α)* is the reaction model.

The temperature dependence of the reaction rate constant can be represented in the Arrhenius form:

(4) $$k\left(T\right)=A\cdot exp\left(-\frac{E_{a}}{RT}\right)$$

In this work, we briefly estimated changes in activation energy during curing for formulation 2 and fitted the reaction model. The kinetic research was based on non-isothermal DSC measurements consisting of three runs with different temperature rates: 5 °C/min, 10 °C/min and 20 °C/min. We used two approaches: model-free isoconversional (Friedman method) and model fitting methods. All calculations were performed in special software, ThermoKinetics 3.1, provided by Netzsch (Selb, Germany).

**Figure 11:**
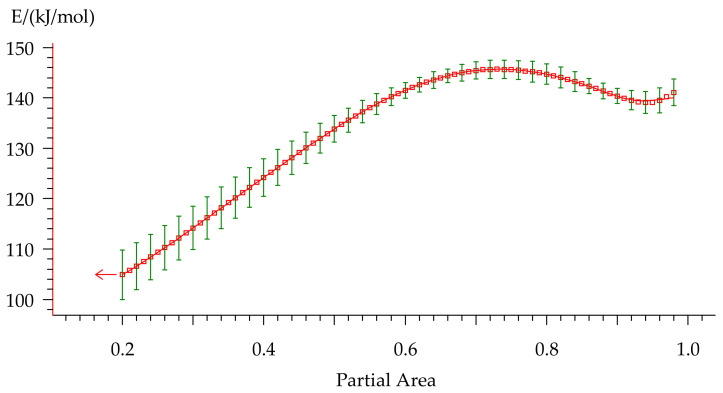
The E_a_-dependency for non-isothermal curing of formulation 2 on the degree of conversion.

Activation energy was calculated by the Friedman method [44] consisting of plotting the dependence (5) of the logarithmic Equation (6), where the slope of the linearized curve provided the activation energy. The resulting dependency is represented in Figure 11.

(5) $$ln{\left(\frac{d\propto }{dt}\right)}_{\propto }-\left(1/T\right)$$

(6) $$ln{\left(\frac{d\propto }{dt}\right)}_{\propto }=ln\left[Af\left(\propto \right)\right]-\frac{E_{a}}{RT}$$

Activation energy varies from 105 to 148 kJ/mol during the curing process that indicates multiple-stage cure kinetics. This type of E_a_-dependency could describe either two parallel independent reactions or two competitive reactions. Presence of the peak contributes to independent reactions. However, a constant rise of E_a_ is usually attributed to competitive reactions. Furthermore, a monotonic change in the peak area that is observed with variation of the heating rate (Table 4) is sufficient evidence of the presence of competitive reactions. As for the small rise of E_a_ at the final stage of curing, we suppose that it could be related to the diffusion control because this behavior is quite typical for this process and was discussed in many works [41,45,46].

In the case of the model fitting approach, we did not obtain an accurate approximation using single-step modeling. This fact is well correlated with the abovementioned results of the Friedman method. Therefore, we decided to separate peaks, calculate their kinetic parameters separately and, after that, combine them into a united two-step scheme model. This method is well discussed by Tikhonov in his study [47].

**Figure 12:**
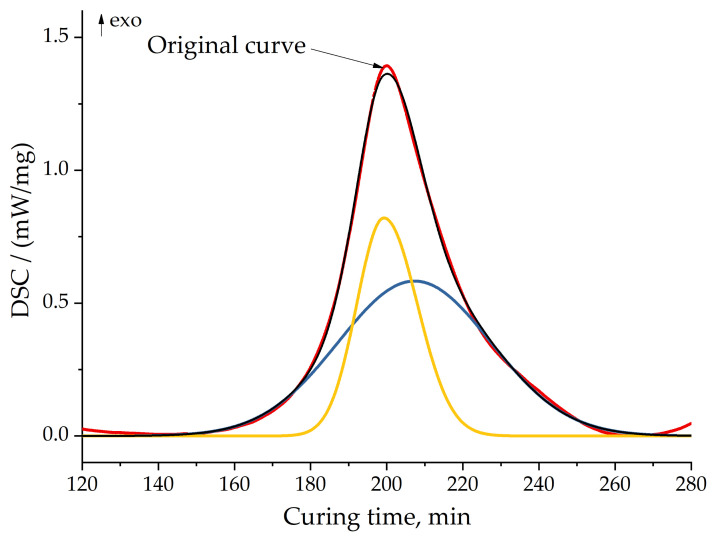
Peak separation for formulation 2 at 10 °C/min. Red line—original curve; yellow and blue lines—separated peaks; black line—model (sum) of separated peaks.

**Figure 13:**
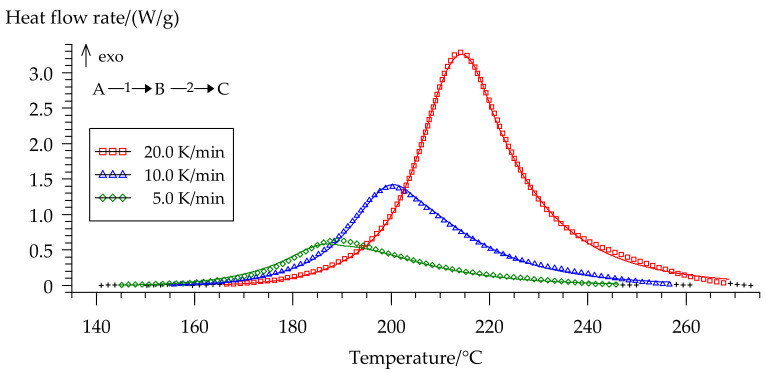
Comparison of experimental (symbols) and calculated (full lines) DSC curves for formulation 2: (□) 20 °C/min, (Δ) 10 °C/min, (◊) 5 °C/min.

We separated peaks in Fityk software (Institute of High Pressure Physics, Warsaw, Poland). A typical graphic of separated peaks for formulation 2 is presented in Figure 12. The attempt to utilize the competitive reaction scheme, predicted by isoconversional analysis, did not succeed. Only the usage of the consecutive reaction scheme resulted in a quite accurate model of curing. Figure 13 shows the final model fitting for composition 2.

The most appropriate type of reaction for two steps appeared to be the extended Prout–Tompkins model:

(7) $$f\left(\propto \right)={\left(1-\propto \right)}^{n}\propto^{m}$$

(8) $$\frac{d\propto }{dt}=A\cdot \exp\left(\frac{-E_{a}}{RT}\right){\left(1-\propto \right)}^{n}\propto^{m}$$

All parameters for the model are presented in Table 5.

A brief kinetics study revealed the complex nature of the curing process of modified composition 2 and proved that this process is not a single step one. The resulting kinetic model of curing was built using a two-step scheme of consecutive reactions and extended Prout–Tompkins model. These facts may indicate that the formation of new bonds between monomers can occur with the participation of different reaction sites both in the aromatic rings of the diphenol and amine residues, and probably the aromatic residue in arylaminophosphazene as well.

### 3.5. Flammability

To estimate the flammability of the resultant cured samples, we used the UL-94 standard.

The content of more than 10 pbw of FR-mt in the composition allows us to achieve the V-0 flammability category of the UL-94 standard. Achievement of the highest category of flame resistance is ensured both by increasing the content of phenyl residues in the composition and by the synergistic effect of phosphorus and nitrogen introduced into the mixture. The results are shown in Table 6.

## 4. Conclusions

From the example of the compositions based on bis(3-(m-tolyl)-3,4-dihydro-2H-1,3-benzoxazine (BA-mt), it was found that hexakis-(3-methylphenylamino)cyclotriphosphazene catalyzes polymerization and, at a content of 10 parts by weight or more, increases the fire resistance rating to the UL-94 V-0 category. The addition of 10 pbw decreases the onset temperature of curing from 222.0 °C for neat BA-mt to 205.9 °C. Due to this reduction, it became possible to proceed to complete curing at temperatures up to 180 °C. DSC curves of cured samples revealed the presence of some undissolved particles of PN-mt in compositions, which could be one of the reasons for the dramatic decrease in glass transition temperature from 206.5 °C (formulation 1) to 160.1 °C (formulation 4). However, we suppose that this disadvantage could be managed. The study of the kinetics of the curing process revealed signs of two competing reactions in the curing of all compositions and of the neat monomer, which probably relate to the phenolic and amine aromatic reactive sites.

## Figures and Tables

**Table 1:** Formulation of mixtures in parts by weight (bpw).

Formulation Number	BA-mt ^1^, pbw	PN-mt ^2^, pbw
1	100	0
2	100	10
3	100	20
4	100	30

^1^ bis(3-(m-tolyl)-3,4-dihydro-2H-1,3-benzoxazine, ^2^ hexakis-(3-methylphenylamino)cyclotriphosphazene.

**Table 2:** Elemental composition of PN-mt.

	C, %	H, %	N, %	Cl, %	P, %
Calculated	65.36	6.27	16.33	0	12.04
Found	65.21	6.21	16.39	0	12.19

**Table 3:** The results of ^1^H NMR spectroscopy of BA-mt.

Sample	Proton Chemical Shifts δ_H_ (ppm)				
	Oxazine Ring		Amine	Diphenol	
	CH_2_N	CH_2_O	CH_3_–	CH_3_–	CH (Ar)
BA-mt	4.56	5.30	2.30	1.57	6.62–7.29

**Table 4:** Characteristic temperatures of DSC curves of uncured and cured samples for formulations 1–4.

Formulation №	Uncured Samples (Figure 6)				Cured Samples (Figure 7)	
	*T_onset_*, °C	*T_peak_*, °C	*T_end_*, °C	Δ*H*, J/g	*T_g(middle)_*, °C	Δ*H_res_*, J/g
**1**	222.0	231.9	242.4	346.2	206.5	23.87
**2**	205.9	220.3	238.2	268.6	164.9	3.18
**3**	195.8	217.0	247.4	260.9	162.3	0
**4**	184.8	209.9	255.7	252.0	160.1	0

**Table 5:** Parameters for resulting two-step model for formulation 2.

Parameter	Optimum Value
logA_1_ [s^−1^]	7.3632
E_1_ [kJ/mol]	80.7604
React. Ord. 1	0.6224
Exponent a_1_	0.5311
Log A_2_ [s^−1^]	13.2443
E_2_ [kJ/mol]	137.0854
React. Ord. 2	2.0102
Exponent a_2_	0.0938
Foll. React. 1	0.1963
Correlation coefficient	0.9993
ΔH (5 °C/min), J/g	278.7
ΔH (10 °C/min), J/g	268.6
ΔH (20 °C/min), J/g	257.5

**Table 6:** Flammability test results according to UL-94 standard.

Formulation №	τ_1_, c	τ_2_, c	τ_3_, c	Σ_τ_, c	UL-94
1	16	19	14	49	V-1
2	5	6	9	20	V-0
3	3	5	7	15	V-0
4	1	4	5	10	V-0

## Data Availability

The data presented in this study are available on request from the corresponding author.
